# A Private Nurse's Earnings

**Published:** 1920-05-29

**Authors:** 


*To the Editor of* The Hospital.

### A Private Nurse's Earnings.

*To the Editor of* The Hospital.

Sir,—I do not agree that I confuse my figures by adding the cost of board, lodging, laundry provided by patients to a nurse's expenses rather than income. Income consists of payments actually received to be spent as one wishes. Nor do I agree that the cost of same amounts to £2 weekly. I should put the figure nearer 25s. at the most, in many cases less. Usually the only additional expenditure to the household accounts caused by the temporary residence of a nurse is for her food and laundry. In some cases the accommodation and food provided are luxurious. In many cases the accommodation is most uncomfortable, and in some cases the food provided is inadequate. Moreover, I would point out that a nurse needs mufti, underwear, boots, shoes, travelling expenses, and many other requirements for fifty-two weeks, not twelve only. I keep a very modest wardrobe, have few amusements, take only short holidays, and, with the exception of tailor-mades, make nearly all my own clothes. Briefly, though a private nurse's fees are higher, the increase is not commensurate with the increased cost of living. My personal expenditure amounts to about £45 a year.

I remain, Sir, yours faithfully,

(Signed) "One Who Knows."

[" One Who Knows" own figure for board, light, and fire was £8 per month.—Ed. T.H.]

